# Different cytokine and chemokine profiles in hospitalized patients with COVID-19 during the first and second outbreaks from Argentina show no association with clinical comorbidities

**DOI:** 10.3389/fimmu.2023.1111797

**Published:** 2023-02-01

**Authors:** Laura Almada, Sofía Carla Angiolini, Nicolás Daniel Dho, Jeremías Dutto, Yamila Gazzoni, Clarisa Manzone-Rodríguez, Constanza Marín, Nicolás Eric Ponce, Daniela Soledad Arroyo, Juan Nahuel Quiróz, Pablo Iribarren, Fabio Marcelo Cerbán, Gabriel Morón, María Carolina Amezcua Vesely, Laura Cervi, Laura Silvina Chiapello, Laura Fozzatti, Paula Alejandra Icely, Mariana Maccioni, Carolina Lucia Montes, Claudia Cristina Motrán, María Cecilia Rodríguez-Galán, Cinthia Carolina Stempin, María Estefanía Viano, Cristian Mena, Mariana Bertone, Claudio Daniel Abiega, Daiana Escudero, Adrián Kahn, Juan Pablo Caeiro, Belkys Angélica Maletto, Eva Virginia Acosta Rodríguez, Adriana Gruppi, Claudia Elena Sotomayor

**Affiliations:** ^1^ Departamento de Bioquímica Clínica, Facultad de Ciencias Químicas, Universidad Nacional de Córdoba, Córdoba, Argentina; ^2^ Centro de Investigaciones en Bioquímica Clínica e Inmunología (CIBICI), Consejo Nacional de Investigaciones Científicas y Técnicas (CONICET), Córdoba, Argentina; ^3^ Instituto Universitario de Ciencias Biomédicas de Córdoba (IUCBC), Hospital Privado Universitario de Córdoba, Córdoba, Argentina

**Keywords:** COVID-19, SARS-CoV-2, comorbidities, cytokine storm, first infection wave, second infection wave, mortality, hypertension

## Abstract

**Background:**

COVID-19 severity has been linked to an increased production of inflammatory mediators called “cytokine storm”. Available data is mainly restricted to the first international outbreak and reports highly variable results. This study compares demographic and clinical features of patients with COVID-19 from Córdoba, Argentina, during the first two waves of the pandemic and analyzes association between comorbidities and disease outcome with the “cytokine storm”, offering added value to the field.

**Methods:**

We investigated serum concentration of thirteen soluble mediators, including cytokines and chemokines, in hospitalized patients with moderate and severe COVID-19, without previous rheumatic and autoimmune diseases, from the central region of Argentina during the first and second infection waves. Samples from healthy controls were also assayed. Clinical and biochemical parameters were collected.

**Results:**

Comparison between the two first COVID-19 waves in Argentina highlighted that patients recruited during the second wave were younger and showed less concurrent comorbidities than those from the first outbreak. We also recognized particularities in the signatures of systemic cytokines and chemokines in patients from both infection waves. We determined that concurrent pre-existing comorbidities did not have contribution to serum concentration of systemic cytokines and chemokines in COVID-19 patients. We also identified immunological and biochemical parameters associated to inflammation which can be used as prognostic markers. Thus, IL-6 concentration, C reactive protein level and platelet count allowed to discriminate between death and discharge in patients hospitalized with severe COVID-19 only during the first but not the second wave.

**Conclusions:**

Our data provide information that deepens our understanding of COVID-19 pathogenesis linking demographic features of a COVID-19 cohort with cytokines and chemokines systemic concentration, presence of comorbidities and different disease outcomes. Altogether, our findings provide information not only at local level by delineating inflammatory/anti-inflammatory response of patients but also at international level addressing the impact of comorbidities and the infection wave in the variability of cytokine and chemokine production upon SARS-CoV-2 infection.

## Introduction

Coronavirus disease 2019 (COVID-19) was initially defined as an atypical pneumonia caused by a novel virus identified as Severe Acute Respiratory Syndrome Coronavirus-2 (SARS-CoV-2) (1). Early on, COVID-19 reached pandemic proportions and to date, more than five hundred and seventy-five million people have been infected and more than six million have died due to this disease (2). COVID-19 clinical manifestations range from mild to severe with a small percentage of patients developing pneumonia and respiratory failure, which may be later complicated by multiorgan dysfunction with acute respiratory distress syndrome (ARDS), shock and death (3–5). Is not fully established why infection with the same virus results in such a spectrum of disease severity; however, current evidence points to the relevance of inter-individual variability of the immune system response and/or predisposing risk factors (6). Insufficient type I interferons (IFNs) response during the first few days of infection is associated with severe COVID-19, underlining the impact of mounting weak anti-viral response that results in impaired viral clearance (7). Also, dysfunctional activation of the immune system that leads to exacerbated production of immune mediators and profound abnormalities in the lymphoid compartment are related with the severity and mortality of SARS-CoV-2 infection (8, 9). Uncontrolled production of cytokines, chemokines, and other inflammatory soluble mediators, also called “cytokine storm”, has been reported as the most mortality related complication that can occur during SARS-CoV-2 infection (10–12). Indeed, the “cytokine storm” has been proposed as a driver of inflammation that is thought to be central to the pathogenesis of severe COVID-19. In addition, predisposing risk factors such as age over 60, male sex and several pre-existent comorbidities have been associated with a worst outcome (13–15), although a link between age, sex, comorbidities, and production of inflammatory/anti-inflammatory mediators has not been clearly established.

Since March 2020, several studies on “cytokine storm” in COVID-19 patients from all over the world have been published (16–18). These studies presented data mostly from the first wave of the outbreak and reported highly variable cytokine profiles particular to different geographical regions where the studies were conducted (19, 20). Likely, the variability was a consequence of different Variants of Concern (VOC) circulation of as well as of population demographics, local sanitary strategies, and health system resilience. In this context, information about the production of soluble mediators in association to SARS-CoV-2 infection outcomes at defined geographical locations may identify particular predictors of disease severity at local population level.

Argentina reported its first COVID-19 case on March 3rd, 2020, and showed different infection waves, with the first two peaking in October 2020 and May 2021 (21). In the present study, we investigated serum concentrations of thirteen soluble mediators, including cytokines and chemokines, in patients with moderate and severe COVID-19 hospitalized in Córdoba, the second most populated city in Argentina, during the first and second infection waves. Comparison between both infection waves highlighted that patients recruited during the second wave were younger and showed less concurrent comorbidities than those from the first outbreak. We determined that concurrent pre-existing comorbidities had an irrelevant contribution to the levels of systemic cytokines and chemokines in COVID-19 patients. Finally, we identified certain parameters such as Interleukin (IL)-6 concentration, C reactive protein (CRP) level and platelet count that allowed to discriminate between death and survival in patients hospitalized with severe COVID-19 only during the first wave. Our data provide incremental information to deepen our understanding of COVID-19 pathogenesis by linking demographics of a COVID-19 cohort with systemic concentrations of cytokines and chemokines, presence of comorbidities and different disease outcomes.

## Methods

### Ethics statement

This study was approved by the “Registro Provincial de Investigación en Salud (RePIS)” (Provincial Registry of Health Research), Córdoba, Argentina under number 4039 and by the Institutional Review Board (IRB) of Hospital Privado Universitario de Córdoba (HPUC), Córdoba, Argentina. Patients enrolled in the study provided written informed consent prior to participation in accordance with the Declaration of Helsinki and data were protected in accordance with Argentine law N° 25.326. Patients included in this study were recruited during the first and second waves of SARS- CoV-2 pandemic in Córdoba, Argentina between October-December 2020 and February-June 2021, respectively (21, 22). In the first wave, the B.1.499 and N.3 lineages were the most common circulating strains (23), while in the second one the gamma, lambda and alpha SARS-CoV-2 variants were predominant, followed by non-VOC non-VOI variants (24).

### Participants

Diagnosis of SARS-CoV-2 infection was made by a SARS-CoV-2 nucleic acid amplification test following guidelines of “Ministerio de Salud” from Argentina. Reverse transcriptase polymerase chain reaction (RT-PCR) (PerkinElmer^®^, Massachusetts, U.S) was performed in nasal and pharyngeal swab samples (21). Only hospitalized patients were recruited (n=118) and classified as moderate or severe COVID-19 according to clinical parameters (25, 26).

Data related to age, gender, pre-existent comorbidities (arterial hypertension, diabetes, overweight, obesity, dyslipidemia, asthma, hypothyroidism, heart disease and any other non-rheumatic and -autoimmune chronic underlying disease) discharge or death, were collected in a database updated daily. The patients did not receive specific anti-inflammatory therapies previous SARS-CoV-2 infection/hospitalization. A cohort of age-matched healthy volunteers (n=24) were recruited and included as healthy control (HC). The healthy individuals recruited for this study had no history of chronic pathologies and showed no symptoms compatible with any infectious process in the last month before sample collection. A single cohort of healthy individuals that covered the age ranges of COVID-19 patients from the first and second waves was used for comparison. Blood samples from HC were obtained at the beginning of the study, during the first wave. HC had no previous history of SARS-CoV-2 infection diagnosed either by positive SARS-CoV-2 nucleic acid amplification test, compatible symptoms, or epidemiological close-contact criteria (21, 25). None of the participants, including HC, received a COVID-19 vaccine before their inclusion in this study.

### Samples

Blood samples from HC and COVID-19 patients, at the time of hospitalization, were collected by venipuncture into BD Vacutainer^®^ SST™ tubes for serum collection. Serum aliquots were stored at –80°C until cytokine multiplex analysis was performed. Blood routine tests (neutrophil and platelet counts, and serum CRP concentration) were performed at the medical laboratory of HPUC. All the samples were analyzed in the same machines.

### Quantification of cytokines and chemokines

Simultaneous quantification of thirteen proteins in serum from COVID-19 patients and HC was performed using a bead-base multiplex assay by flow cytometer (LEGENDplexTM Human Anti-Virus Response Panel, Cat. # 740390, Biolegend). Concentration (pg/ml) of the following proteins was determined: IFNs type 1 (IFN-β and IFN-α2), IFN type 2 (IFN-γ), IFNs type 3 (IFN-λ1 or IL-29 and IFN-λ2/3 or IL-28A/B), IL-1β, IL-6, IL-8, IL-12p70, IL-10, tumor necrosis factor (TNF), granulocyte macrophage colony stimulating factor (GM-CSF) and IFN-γ-inducible protein 10 (IP-10). Serum samples and standards were run in duplicates in plates according to the manufacturer’s instructions with some modifications. Briefly, a mix (50:50) of serum and Assay Buffer or standards and Matrix B were incubated with mixed beads, washed, and incubated with biotinylated detection antibodies and PE-conjugated streptavidin. Two pools of sera from HC (male and female) were included in each measurement for quality assurance purposes (data not shown). The samples were transferred from plate to tube and read using a FACSCanto II™ flow cytometer and data analyzed with LEGENDplexTM Data Analysis Software. Cytokine concentration was normalized using logarithm scale with base 10.

### Statistical analysis

Prior the statistical analysis, a normality test (Shapiro–Wilk test) was used to determine the normal distribution of the datasets. Descriptive analysis of categorical variables was expressed as absolute and relative frequencies and numerical variables were expressed as mean (± standard error of mean, SEM). Chi-Square was used to test differences in frequency of mortality and comorbidities between different categories of patients. Association between sex, COVID-19 severity and mortality was evaluated using odds ratio (OR) (27) and 95% confidence interval (CI). Further specifications for the statistical test used are detailed in each figure. Statistical and graphical data analysis were done with GraphPad Prism version 7.0 for Windows (GraphPad Software, San Diego, CA, USA) and R studio version R-4.0.2 (28).

Multivariate logistic regression was conducted to explore predictors of mortality (IL-6, IL-8 and IFN-α2) by estimating the 95% CI. Receiver Operating Characteristics (ROC) curve analysis was done to evaluate sensitivity and specificity of the model. Performance of the model was evaluated with the area under the curve (AUC) derived from (ROC) analysis. For every test, p-value lower than 0.05 was considered statistically significant.

## Results

### Demographic characteristics in patients with COVID-19 were different in the first and second waves

**Figure 1:**
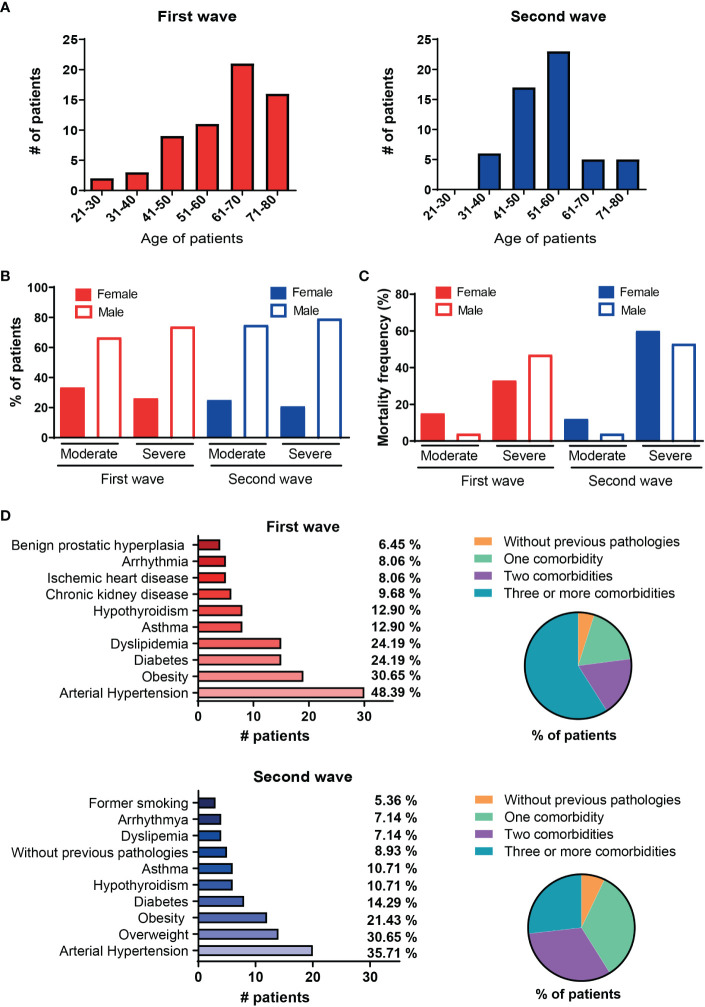
Demographic and clinical characteristic of hospitalized COVID-19 patients from the first and the second waves in Argentina. **(A)** Number of patients distributed by age range during the first (red, n=62) or second waves (blue, n=56). **(B)** Gender distribution of patients from the first and second waves stratified according to clinical disease severity in moderate and severe. **(C)** Frequency of occurrence of death (mortality) by gender of patients from the first and second waves stratified according to clinical disease severity in moderate and severe. **(D)** Frequency of comorbidities in patients during the first (left upper chart) and second (left lower chart) waves, pie charts on the right show frequency of concurrent comorbidities in patients from each wave of infection.

During the first and second outbreaks of SARS-CoV-2 infection in the city of Cordoba, in the central region of Argentina, a total of 118 patients hospitalized with COVID-19 and 24 HC were recruited in this clinical study. Demographic characteristics showed that most of COVID-19 patients recruited during the first wave were in the age range of 61-80 while the cohort from the second wave was younger, with patients aged between 41-60 (**Figure 1A**). Mean of age of patients from the second wave was significantly lower than in the patients from the first one (p<0.05, data not shown). According to clinical parameters, patients were categorized in moderate and severe as described in Methods. Males represented the majority of patients in our cohorts with moderate and severe disease, both in the first (moderate: 67%; severe: 74%, OR: 1.31, 95% CI 0.4-4.5) and in the second wave of the pandemic (moderate: 75%; severe: 79%, OR: 1.25, 95% CI 0.3-4.9) (**Figure 1B**). Evaluation of mortality frequency showed that death rate was lower during the first and second waves in males than females within the group with moderate disease (**Figure 1C**). As expected, frequency of deceased patients increased in the cohort of severe disease in both waves (total severe patients versus total moderate patients, p<0.005 and p<0.0005 for first and second wave, respectively, data not shown). Male patients with severe disease from the first wave showed higher mortality risk than their female counterparts (OR: 1.49, 95% CI: 0.2-4.1). In contrast, mortality risk in male patients with severe disease was reduced in the second wave (OR: 0.86, 95% CI: 0.8-4.1). Global mortality was 51% and 60% in the first and second infection waves, respectively.

The most common pre-existent comorbidities present in the cohort from the first wave were hypertension (48.39%), obesity (30.65%), diabetes (24.19%) and dyslipidemia (24.19%) while hypertension (35.71%), overweight (30.65%), obesity (21.43%) and diabetes (14.29%) were predominant in patients recruited during second wave (**Figure 1D**). The percentage of patients in each outbreak that have none, one, two, three or more comorbidities are shown in a pie chart on the right. Remarkably, 54% of patients had three or more comorbidities in the first wave while this percentage decreased to 26% during the second wave.

### Differential intensity in the signatures of systemic cytokines and chemokines in patients recruited during the first and the second waves of COVID-19

**Figure 2:**
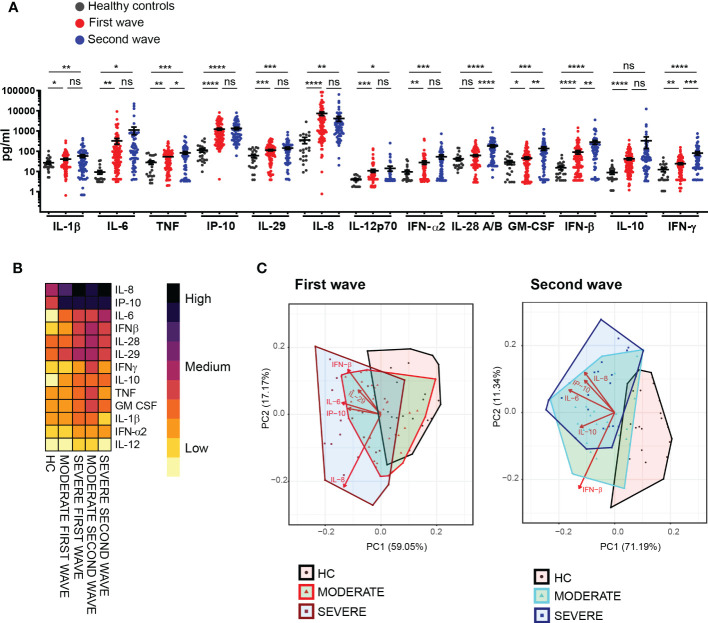
Different intensity in the signatures of systemic cytokines and chemokines in patients recruited during the first and second waves of COVID-19. **(A)** Serum concentration of 13 cytokines/chemokines quantified by LEGENDplexTM in samples from healthy controls **(HC)** (black, n=24) and from COVID-19 patients from the first (red, n=102) and the second wave (blue, n=66). Dots show individual measurements and black line show mean concentration of each analyte ± SEM. Unpaired t test with Welch’s correction was used for statistical analysis. ****P < 0.0001, ***P < 0.001, **P < 0.01 and *P < 0.05; ns: not significant. **(B)** Heat map depicting average concentration of serum cytokines/chemokines in HC and patients from the first and the second waves stratified as moderate and severe according to clinical disease severity. **(C)** PCA biplots average cytokines/chemokines systemic concentration from HC and patients from the first (left plot) and the second (right plot) waves stratified as moderate and severe according to clinical disease severity.

To compare features of the “cytokine storm” in the cohort of patients from the first and second waves, we evaluated serum concentrations of thirteen cytokines and chemokines related to inflammation and viral control using LEGENDplexTM Human Anti-Virus Response Panel. As depicted in **Figure 2A**, patients infected during the first wave showed significantly increased serum concentration of all the cytokines/chemokines measured when compared to HC, with the only exception of IL-28 that showed similar concentration in the serum of patients and controls. Similarly, the cohort of patients infected during the second wave exhibited higher serum concentration of all the analytes evaluated except for IL-10 that showed no differences between infected patients and controls. We detected five cytokines (TNF, IL-28, GM-CSF, IFN-β and IFN-γ) which concentrations were significantly higher in serum of patients infected in the second wave as compared to those infected in the first one. Interestingly, three out of these five cytokines were type I (IFN-β), type II (IFN-γ) and type III (IL-28) IFNs that are closely associated to anti-virus immunity.

We next aimed to determine whether the intensity of cytokines/chemokines response was associated with clinical severity. To accomplish this goal, and as a first approach for visualization, we generated a heatmap with the average level of each cytokine/chemokine measured in patients serum within each infection wave, stratified into moderate and severe (**Figure 2B**). As shown, general cytokine/chemokine profiles were similar among all COVID-19 patients of our cohort, independently of clinical severity and wave of infection. However, some differences, particularly in intensity of the response, were identified. In this regard, patients infected in the second wave showed a signature of cytokine/chemokine production with an overall increased magnitude as compared to those infected in the first wave. Furthermore, within the groups of each wave, patients categorized as severe showed differences in the intensity of certain cytokines and chemokines production that were particular to each wave. Thus, during the first infection wave, IL-8 was significantly (p<0.05) increased predominantly in patients with severe disease versus those with moderate COVID-19 and a tendency in the same direction was observed for IL-6, IL-10 and IFN-α2. This profile was not observed in the cohort from the second wave that showed elevated concentration of these four cytokines independently of disease severity. Distinctively, a significantly reduced production of TNF and IFN-γ (p<0.05) was observed in patients with severe disease from the second wave.

Finally, we performed a principal component analysis (PCA) of all the analytes determined in sera from patients and controls with moderate and severe disease within the cohorts from the first and second waves (**Figure 2C**). As shown in the PCA biplots, principal component 1 (PC1) together with principal component 2 (PC2) explained 76.22% of the total variability among clinical groups in the first wave and 82.53% in the second wave. Analysis in the clinical groups from the first wave indicated that the signature of serum cytokines and chemokines in both infected groups and controls overlapped partially but also showed a segregation trend from controls to patients with moderate disease and to patients with severe disease. Differently, the results for the second wave cohort showed a complete segregation of the infected groups away from controls that almost completely depended on PC1. Clusters of patients with moderate and severe disease overlapped but showed certain segregation at expense of PC2 (**Figure 2C**). We further focused to the analytes that accounted for these results and determined that the top five variables contributing to PC1 and PC2 included IL-8, IP-10, IL-6, IFN-β and IL-29 for the first wave cohort and IL-8, IP-10, IL-6, IFN-β and IL-10 for the second wave cohort. The biplots show the vectors for each analyte indicating the direction and length (as measure of magnitude) of the contribution.

### Conserved signature of systemic cytokines and chemokines in COVID-19 patients with different comorbidities

With the aim of evaluating if the profile of soluble mediators quantified in hospitalized COVID-19 patients was influenced by any of the comorbidities most frequent in our cohort, we analyzed the profile of cytokine/chemokine expression in particular groups of patients segregated according to a previous diagnosis of hypertension, diabetes, and obesity.

**Figure 3:**
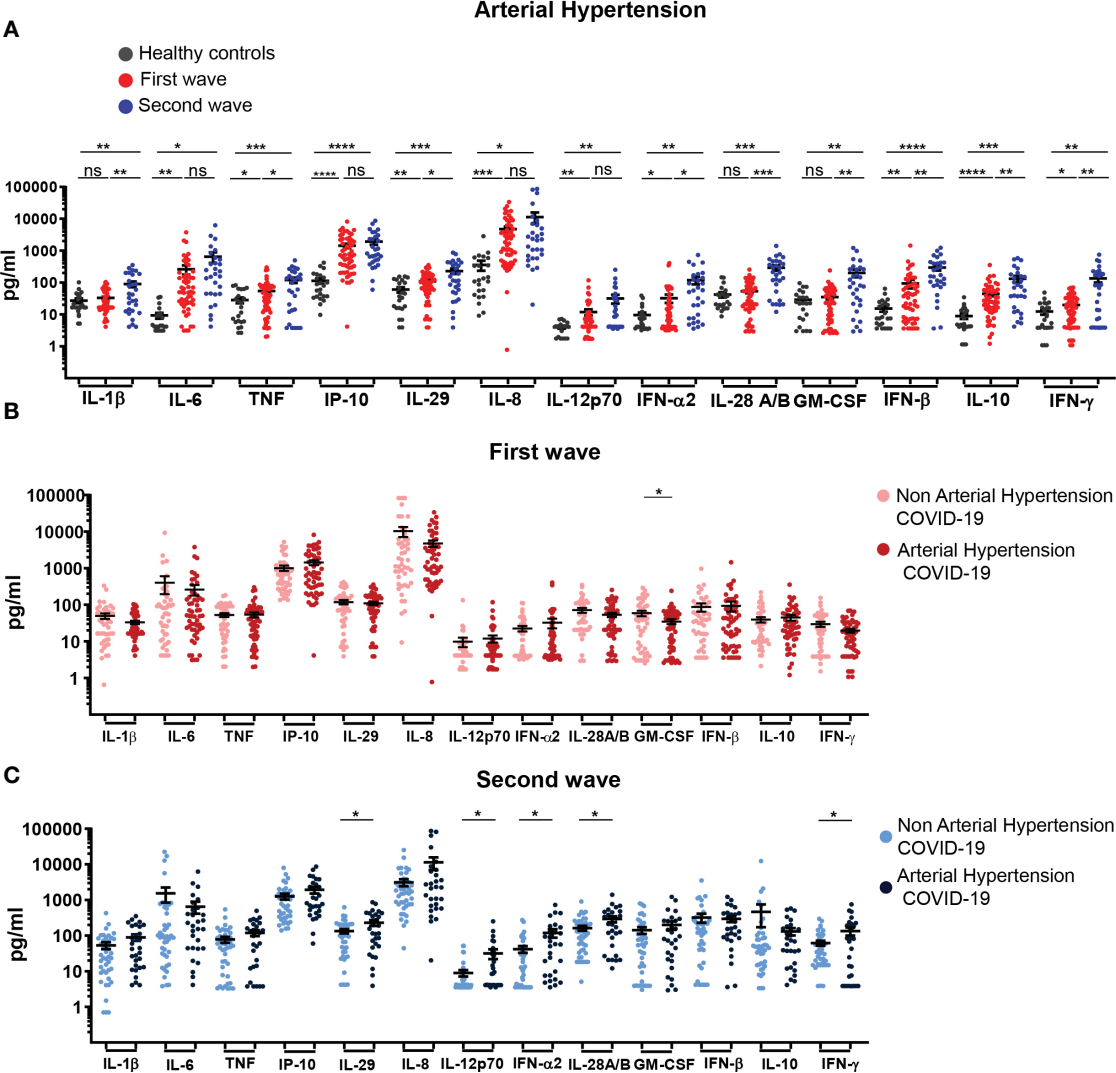
Profile of systemic cytokines and chemokines in hospitalized COVID-19 patients with arterial hypertension recruited during the first and second waves. **(A)** Serum concentration of cytokines/chemokines quantified in samples from HC (black, n=24) and COVID-19 patients with arterial hypertension recruited during the first (red, n=55) and second (blue, n=29) waves. **(B)** Serum concentration of cytokines/chemokines determined in samples of COVID-19 patients without (light pink, n=47) or with (dark pink, n=55) arterial hypertension of first wave. **(C)** Serum concentration of cytokines/chemokines determined in samples of COVID-19 patients without (light blue, n=37) or with (dark blue, n=29) arterial hypertension of the second wave. The scatter plots in **(A-C)** show individual measurements (dots) and the concentration mean of each analyte as black line ± SEM. For statistical analyses Unpaired t test with Welch’s correction was used (****P < 0.0001, ***P < 0.001, **P < 0.01 and *P < 0.05; ns: not significant).

Hypertensive COVID-19 patients from both infection waves had significantly higher serum concentration of IL-6, TNF, IP-10, IL-29, IL-8, IL-12p70, IFN-α2, IFN-β, IL-10 and IFN-γ in comparison with controls (**Figure 3A**). Hypertensive patients from the second but not from the first wave also showed elevated IL-1β, IL-28 and GM-CSF versus controls. From these cytokines, IL-1β, TNF, IL-29, IFN-α2, IL-28, GM-CSF, IFN-β, IL-10 and IFN-γ exhibited increased level in patients with hypertension in the second outbreak in comparison to those from the first one. Comparison of serum cytokine/chemokine concentration in patients with or without hypertension within the cohort from the first wave showed no significant differences with the only exception of GM-CSF (**Figure 3B**). A similar analysis within the cohort from the second wave demonstrated that hypertensive patients presented a significant increase in the concentration of cytokines associated to an IFNs response and Th1 profile such as IL-12p70, IL-28, IL-29, IFN-α2 and IFN-γ in comparison to non-hypertensive patients (**Figure 3C**). The evaluation of mortality showed non-significant differences reaching 23.33% of hypertensive versus 18.75% of non-hypertensive patients during the first wave and 27.78% versus 25.00% respectively in the second outbreak.

**Figure 4:**
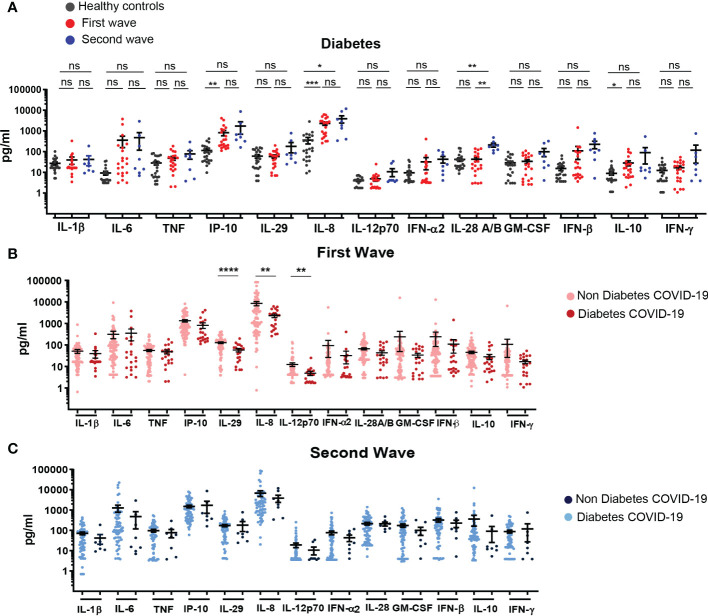
Profile of systemic cytokines and chemokines in hospitalized COVID-19 patients with diabetes recruited during the first and second infection waves. **(A)** Serum concentration of cytokines/chemokines quantified in samples from HC (black, n=24) and COVID-19 patients with diabetes recruited during the first (red, n=23) and second (blue, n=8) waves **(B)** Serum concentration of cytokines/chemokines in samples of COVID-19 patients without (light pink, n=81) or with (dark pink, n=21) diabetes recruited during the first wave. **(C)** Serum concentration of cytokines/chemokines determined in samples of COVID-19 patients without (light blue, n=58) or with (dark blue, n=8) diabetes recruited during the second wave. The scatter plots in **(A-C)** show individual measurements (dots) and the concentration mean of each analyte as black line ± SEM. For statistical analyses Unpaired t test with Welch’s correction was used (****P < 0.0001, ***P < 0.001, **P < 0.01 and *P < 0.05; ns: not significant).

In diabetic patients infected during the first and second waves, we observed that concentration of most of the evaluated cytokines/chemokines were similar to those of controls, with the exception of IP-10, IL-8 and IL-10 and of IL-8 and IL-28 that were increased in diabetic COVID-19 patients from the first and second wave, respectively, when compared to controls (**Figure 4A**). Interestingly, in diabetic COVID-19 patients from the first wave, IL-29, IL-8 and IL-12p70 were decreased compared to controls (**Figure 4B**). No significant differences in serum cytokine/chemokine concentration were detected when comparing COVID-19 patients from the second wave with or without diabetes (**Figure 4C**). The percentages of mortality in patients with or without diabetes were similar, showing non-significant differences during the first (20.00% versus 21.28, respectively) and second wave (25.00% versus 27.08%, respectively).

**Figure 5:**
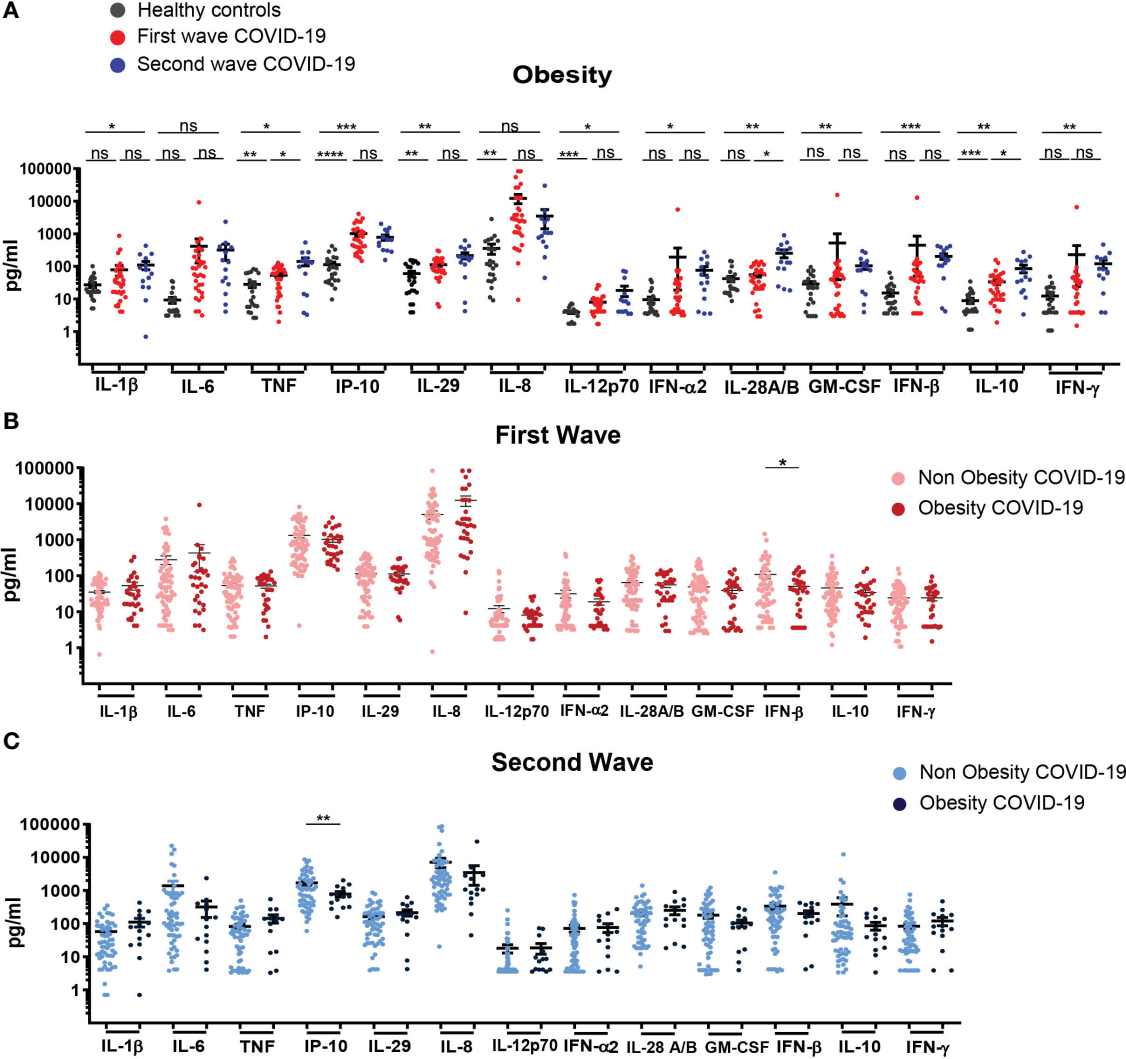
Profile of systemic cytokines and chemokines expression in in hospitalized COVID-19 patients with obesity recruited during the first and second infection waves. **(A)** Serum concentration of cytokines/chemokines in samples from HC (black, n=24) and hospitalized COVID-19 patients with obesity recruited during the first (red, n=32) and second waves (blue, n=14). **(B)** Serum concentration of cytokines/chemokines in samples of COVID-19 patients without (light pink, n=70) or with (dark pink, n= 32) obesity recruited during the first wave of infection. **(C)** Serum concentration of cytokines/chemokines in samples of COVID-19 patients without (light blue, n=52) or with (dark blue, n=14) obesity during the second wave. The scatter plots in **(A-C)** show individual measurements (dots) and the concentration mean of each analyte as black line ± SEM. For statistical analyses Unpaired t test with Welch’s correction was used (***P < 0.001, **P < 0.01 and *P < 0.05; ns: not significant).

When obese patients from the first and second wave were analyzed in comparison to controls, we observed that patients from the first wave exhibited increased concentration of TNF, IP-10, IL-29, IL-8, IL-12p70 and IL-10 while patients from second wave presented higher levels of almost all mediators evaluated with exception of IL-6 and IL-8 (**Figure 5A**). When comparing the profile of cytokines/chemokines in obese patients from both waves, we established that obese patients from second wave showed higher levels of TNF, IL-28 and IL-10. Remarkably, concentration of cytokines/chemokines in serum of obese and non-obese patients from the first and the second wave were similar; with the exception of IFN-β and IP-10, which were significantly reduced in obese respect to non-obese patients from the first and second waves, respectively (**Figures 5B, C**). The evaluation of mortality in this group of patients showed non-significant differences, reaching 15.79% of obese versus 23.26% of non-obese patients during the first wave and 16.67% versus 29.55% respectively, in the second outbreak.

### Irrelevant contribution of concurrent comorbidities to cytokines and chemokines concentration in serum of COVID-19 patients

**Figure 6:**
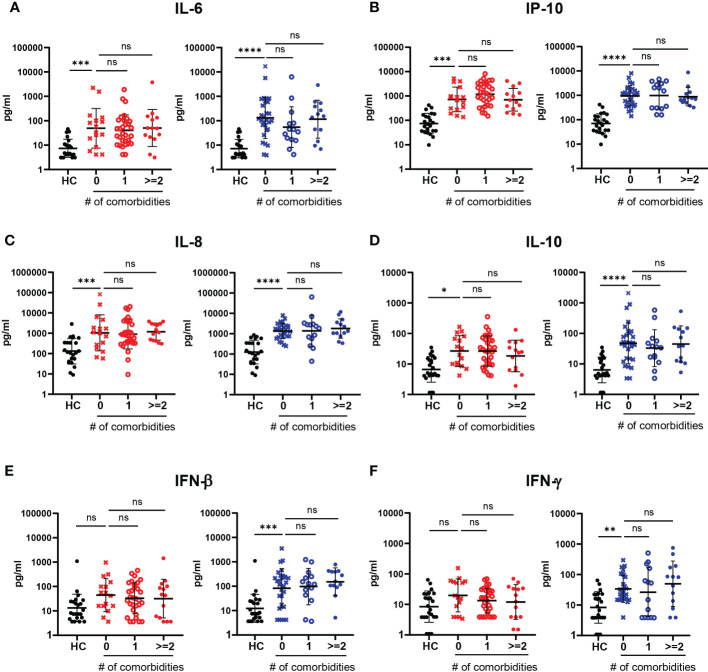
Impact of the number of comorbidities in the serum levels of cytokines and chemokines in hospitalized COVID-19 patients recruited during the first and second infection waves. **(A–F)** Serum concentration of IL-6 **(A)**, IP-10 **(B)**, IL-8 **(C)**, IL-10 **(D)**, IFN-β **(E)** and IFN-γ **(F)** in HC and COVID-19 patients without comorbidities (0, x symbol) and with one (1, ⚬ symbol) or two or more than two (>=2, ● symbol) concurrent comorbidities recruited during the first (red symbols) and the second (blue symbols) waves. The scatter plots in **(A-F)** show individual measurements (dots) and the concentration mean of each analyte as black line ± SEM. For statistical analyses Unpaired t test with Welch’s correction was used (****P < 0.0001, ***P < 0.001,**P < 0.01 and *P < 0.05; ns: not significant).

We next aimed to evaluate whether the presence of concurrent comorbidities in our cohort of hospitalized COVID-19 patients conditioned the production of soluble mediators. For this goal, we performed a comparative analysis of the cytokines/chemokines concentration in serum of hospitalized COVID-19 patients without comorbidities or with one, two or more than two simultaneous comorbidities in comparison to controls, using data from both infection waves. As shown in **Figures 6A–D**, patients without comorbidities exhibited higher level of IL-6, IP-10, IL-8 and IL-10 in comparison with controls. Remarkably, level of these mediators were not further increased by the presence of one, two or more concurrent comorbidities. The same profile was observed during the first and second waves of the outbreak, indicating that the production of cytokines and chemokines triggered by SARS-CoV-2 infection, at least those evaluated in this study, were independent to the number of coexisting diseases present in infected patients.

We also observed that the serum level of IFN-β and IFN-γ were significantly increased in hospitalized COVID-19 patients without comorbidities respect to controls, and the amount of these antiviral mediators were not affected by the presence of multiple comorbidities for patients recruited during the second, but not the first wave (**Figures 6E, F**).

### IL-6, CRP and platelet counts discriminated between death and discharge in patients hospitalized with severe COVID-19 during the first wave

**Figure 7:**
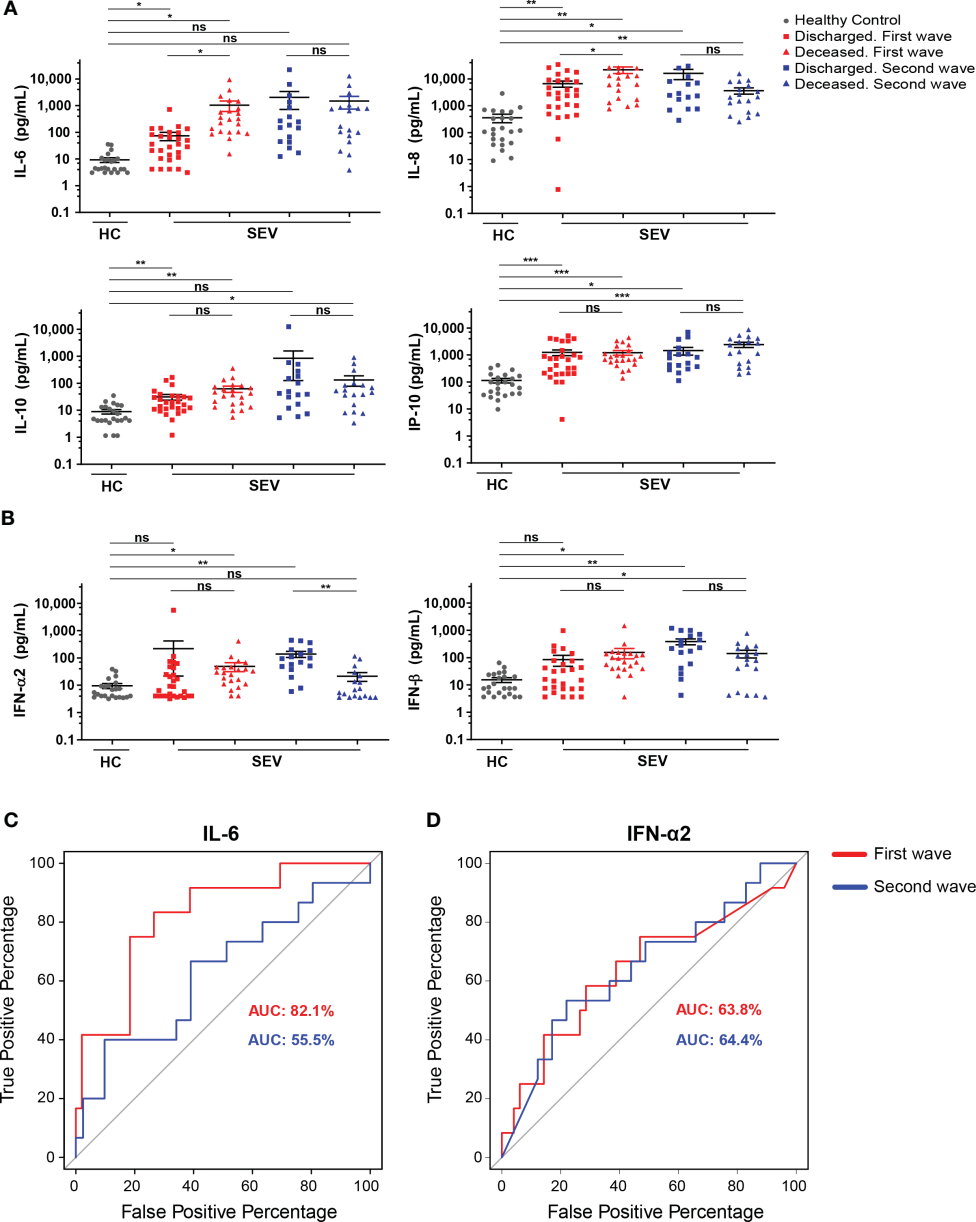
Differences in cytokine and chemokine concentration in hospitalized patients with severe COVID-19 that had different clinical outcome**. (A, B)** Serum concentration of IL-6, IL-8, IL-10 and IP-10 **(A)** and IFN-α2 and IFN-β **(B)** determined in samples from HC (gray) and from discharged and deceased severe COVID-19 patients recruited during first (red) and second (blue) waves. Scatter plots show the individual measurements (dots) and black line show the concentration mean of each analyte. For statistical analyses Unpaired t test with Welch’s correction was used (***P < 0.001, **P < 0.01 and *P < 0.05; ns, not significant). **(C, D)** ROC curve of serum IL-6 **(C)** and IFN-α2 **(D)** concentrations in deceased versus discharged severe COVID-19 patients from the first (blue lines) and the second (red lines) waves. Values of AUC for each wave are shown with the corresponding color code.

In order to identify prognostic factors of COVID-19 outcomes, we initially compared serum concentration of cytokines/chemokines between patients with severe disease that were either discharged or deceased during the two infection waves. Among all cytokines/chemokines with high serum concentration in the whole cohort of COVID-19 patients, we focused on those particularly related to inflammatory/anti-inflammatory responses (**Figure 7A**) and to control of viral infection (**Figure 7B**). In the first set of mediators, we determined that IL-6 and IL-8 presented higher values in deceased versus discharged group during the first wave but not during the second one (**Figure 7A**). In addition, IFN-α2 presented lower values in deceased patients in comparison to discharged (live) patients only during the second wave (**Figure 7B**). Also, a standard set of clinical laboratory studies including CRP, neutrophils and platelet counts were compared between both groups. The results revealed that there were significant differences in CRP level, being higher in the deceased group in both waves. Differently, higher neutrophils and lower platelet counts were observed in those patients who died compared to those who survived during the first, but not the second wave (**Supplementary Figure 1A**).

Next, we attempted to test the potency of these biomarkers in predicting mortality using ROC curve analysis. During the first wave, the AUC of IL-6 was 82.1% (95% CI 0.67-0.97, p=0.02), and the optimum cutoff was 97.3 pg/mL (sensitivity 75.0%, specificity 82.0%) (**Figure 7C**). Of note, the AUC of IL-6 in the second wave fell to 55.5%, losing significance as mortality predictive variable (95% CI 0.48-0.62, p=0.15). Further ROC curve analyses showed that IL-8 (data not shown) and IFN-α2 (**Figure 7D**) did not achieve a significant discriminative power in any of the two waves. In regard to laboratory findings, we calculated that the AUC of CRP was 79.3% (95% CI 0.59-1,00, p=0.005) with an optimum cutoff of 17.40 mg/dL (sensitivity 0.57%, specificity 0.98%) during the first wave while it was 75.8% (95% CI 0.55-0.97, p=0.067) during the second wave (**Supplementary Figure 1B**). In addition, the AUC of platelet count was 71.6% (95% CI 0.50-0.93, p=0.02) with an optimum cutoff of 235,500/μL (sensitivity 81%, specificity 62%) for the first wave and 42.6% (95% CI 0.26-0.59, p=0.29) for the second. Finally, the AUC of neutrophil count was 70.1% (95% CI 0.46-0.95, p=0.053); and 54.9% (95% CI 0.34-0.76, p=0.37) for the first and second waves, respectively.

Altogether, these findings show that some parameters such as IL-6, IL-8, type I IFNs, CRP, neutrophil and platelet counts presented significant differences in patients with severe COVID-19 with different clinical outcomes (deceased versus discharged). Among them, however, the main predictors of death by severe COVID-19 patients were IL-6, CRP and platelet count but only during the first wave.

## Discussion

Since the beginning of SARS-CoV-2 pandemic, a large number of studies have been carried out with the purpose of characterizing and understanding COVID-19 pathophysiology and to provide biomarkers for the prognosis of disease severity (6). While many studies evaluated COVID-19 patients belonging to a single pandemic wave, just a limited number of reports compared patient characteristics between successive pandemic outbreaks. Few studies that compared COVID-19 patients from two first waves mainly focused their analysis on demographic features, predominant pre-existing comorbidities, laboratory data and clinical manifestations together with COVID-19 outcome. These studies were performed in various countries and reported significant variabilities in SARS-CoV-2 infection behavior within different waves (19, 20, 29–31). In this scenario, our study, that compares demographic and clinical features of COVID-19 patients from Argentina during the first two waves of the pandemic and analyzes possible associations with the “cytokine storm” offers added value to the field.

According to predominant international evidence, the first wave of SARS-CoV-2 infection mainly affected elderly people with pre-existing comorbidities. Differently, a significant increase in the number of infected young adults admitted to intensive care unit and a reduction in disease severity was observed during the second wave, particularly in countries of Europe, North America, and Asia (29–35). In agreement with these published data, patients evaluated in this study were older in the first, in comparison to the second outbreak in Argentina. Sex-disaggregated data showed that, within our cohort of hospitalized patients, prevalence of SARS-CoV-2 infection was increased in men versus women in both waves and that men from the first wave exhibited a higher risk of severe disease than women. Although some studies associated female gender with a higher risk for COVID-19 (36), our results agree with reports published during the pandemic describing increased incidence of COVID-19 in men as well as fatal outcomes in this gender particularly during the first wave (37–39).

As reported in other countries, we found that the most frequent pre-existing comorbidities during the first wave were hypertension, obesity, and diabetes (37, 40, 41). Hypertension headed the list of comorbidities in our cohort of patients in both waves highlighting similarities with reports from Brazil (42) but different with other Latin American countries such as Mexico (43) and Peru (44) in which obesity was the predominant comorbidity associated to COVID-19. Somehow expected, and probably as a consequence of their reduced overall age, patients from the second wave showed less concurrent comorbidities than those from the first outbreak.

As described above, our evaluation about the demographic of hospitalized patients infected in the first two COVID-19 waves in Argentina identified differences in age, comorbidities, and clinical outcomes. Furthermore, epidemiological data about the predominant circulating VOC suggested that patients were infected with different variants in each outbreak (21, 22). In these cohorts, we performed a real-time quantification of selected cytokines and chemokines related to viral control and inflammation. This monitoring was aimed to assist in clinical decisions during hospitalization. As a first important conclusion, we observed that hospitalized COVID-19 patients exhibited an increase in systemic concentration in 12 out of 13 cytokines and chemokines evaluated. These results underlie that beyond different demographic features of patients and the circulating VOC, a cytokine storm was induced during both outbreaks of SARS-CoV-2 infection. Of note, qualitative and quantitative differences between cohorts from both waves were detected and further analyzed.

In patients from the first wave, IL-8 was the most increased systemic chemokine, followed by IL-6 and IP-10. IL-6 and IL-8 concentration were higher in deceased than survived patients with severe disease from the first wave. In the second wave cohort, high concentration of these cytokines were also detected, but the levels were similar between deceased and discharged severe patients, showing that the first wave was mainly marked by a pro-inflammatory response. In the second wave, TNF, IL-28, GM-CSF, IFN-β, and IFN-γ reached higher concentrations than those detected in patients from the first wave. Remarkably, three of these cytokines were interferons. Considering type I (IFN-β) and III (IL-28) IFNs as relevant for viral clearance and type II IFN (IFN-γ) as drives for activation of immune cells and mediators (45), our results suggest that patients from the second wave responded with a better protective antiviral response. However, deceased patients from the second wave had lower concentration of IFN-α2 than discharged patients. As insufficient type I IFNs immunity has been linked to life-threatening COVID-19 pneumonia (46), treatments with type I IFNs were proposed as COVID-19 therapy. Supporting this idea, patients treated with IFN-α2b had reduced duration of virus in the upper respiratory tract and IL-6 and CRP systemic concentration (47). However, there is increasing evidence that patients with severe COVID-19 have a robust type I IFNs response (48), which contrasts with the delayed, possibly suppressed, response seen early in infection. In this regard, it has been proposed that type I IFNs might have an important role in exacerbating TNF and IL-1- driven inflammation in progression to severe COVID-19 (48). The contradictory results regarding type I IFNs response in COVID-19 patients might be explained by differences in definitions of disease severity, sampling time points, type of readout (for example, type I IFNs itself or cellular responses to type I IFNs) and location of the response (lung versus systemic) between studies. Altogether, these data suggest that location, timing, and duration of IFNs exposure are critical parameters underlying the success or failure of this kind of therapy for COVID-19.

The rather small difference in signature of systemic cytokines and chemokines between patients from the first and second outbreaks, as mentioned earlier, was more evident when patients from each waves were stratified according to clinical severity. Particular signatures could be established, and each wave presented particular patterns of segregation between groups as demonstrated by PCA analysis. Discrimination was achieved at expense of IL-8, IL-6, IFN-β, IP-10 concentration, showing similarities with the profile of inflammatory mediators described in patients from other regions (49–52). It has been reported that IL-6, IL-8, and IL-10 are associated with systemic inflammation and are increased in patients with COVID-19-related ARDS (53). Besides IL-6, CRP and platelet and neutrophil counts also allowed to discriminate between death and discharge in patients hospitalized with severe COVID-19, but only during the first wave. Altogether, our data demonstrate that severity and mortality during COVID-19 was significantly linked to a pro-inflammatory response only during the first wave.

Research about the mechanisms underlying COVID-19 immunopathology early identified IL-6 as a deleterious factor and proposed this cytokine as one of the main therapeutic targets to be blocked to improve patient survival and outcome. Following this direction, two IL-6 receptor antagonists, tocilizumab and sarilumab have been used in COVID-19 with variable outcomes (54–57). We found that IL-6 was significantly associated with death by COVID-19 only during the first wave leading us to question the role of this cytokine as a single biomarker associated with fatal outcomes. Previous studies in other countries as Spain, Italy, and France reported similar results, showing that patients of second wave had a lower inflammatory component when compared to patients from first wave (58–61). For example, Gelso et al. reported that patients from the second wave in Italy presented lower level of inducible nitric oxide synthase, IL-6 and IL-10, as compared to the corresponding group of the first wave (60). Similar reduction in concentration of multiple cytokines, including IL-6, in COVID-19 patients from the second versus the first wave in Italy were also reported (62). On the contrary, patients from second wave had greater lymphopenia and IL-6 that those of first wave in India (63). These along with our data, suggest that therapeutic blockade of IL-6 biological functions should not be considered a general applicable treatment but rather a personalized strategy. This approach could be contemplated in light of the inflammatory profile of each patient that will be likely influenced by demographic characteristics and the circulating VOC.

To highlight the role of cytokines in COVID-19, an important point was to consider if those treatments, used in autoimmune diseases, aimed at blocking cytokines action affect the susceptibility of patients to SARS-CoV-2 or the evolution of the infection. In this direction, it has been described that patient with plaque psoriasis receiving biologic therapies, do not have higher adverse events or severe complications of the SARS-CoV-2, compared with the general population (64). Another study reported that psoriatic patients on biologic were at higher risk to test positive for COVID‐19 and hospitalized, however, not increased risk of intensive care unit admission or death were found (65). Despite of the apparent controversy, it could be concluded that those treatments focused on blocking the effect of cytokines do not lead to a worse evolution of the infection.

Co-existing diseases such as cardiovascular disease, cancer, diabetes, and others increase the likelihood of severe outcomes in COVID-19 patients by modulating host-viral interactions and immunity, promoting severe infection and death (66). In general, comorbidities most frequently present in patients of our cohort have been linked to an imbalance of effector and regulatory T cells which may reflect a loss of T cell homeostasis (67, 68). In addition, multiple studies have described an association between severe COVID- 19 and an exacerbated pro-inflammatory response (69, 70). Although cytokine release syndrome usually resolves following viral clearance, its persistence led to tissue damage, multiple organ failure and death in critically ill patients (71). Considering this information, we wondered if patients with pre-existing comorbidities responded differentially in terms of cytokine production to SARS-CoV-2 infection. Unexpectedly, we determined that the profiles of systemic cytokine and chemokine responses in COVID-19 patients from both waves were qualitative and quantitatively independent of the type and number of concurrent comorbidities. These data support the hypothesis that pre-existing diseases or risk factors are not critical determinants of inflammatory response features induced after this viral infection. The interpretation of our data in the context of available literature is that an inflammatory response of a relatively high intensity will have a more profound impact in infected patients with pre-existing comorbidities associated to basal organ damage than in those with a healthier background. In this regard, patients with essential hypertension are characterized by endothelial dysfunction and impaired nitric oxide availability secondary to oxidative stress production (72). It has been reported that endothelial dysfunction contributes to severe COVID-19 in combination with dysregulated lymphocyte responses and cytokine networks (73). Ruhl et al. (73) suggested that, besides a strong inflammatory response, severe COVID-19 is driven by endothelial activation and barrier disruption and proposed that recovery depends on the regeneration of endothelial integrity. Then, preceding endothelial dysfunction combined with the direct assault of SARS-CoV-2 on vascular system may result in an enhanced damage as consequence of a conventional inflammatory response and account for the high mortality of COVID-19 patients with pre-existing conditions (74). Although, the frequency of fatal cases was not affected by the presence of hypertension, diabetes or obesity in our cohorts, the fact that most of the hospitalized patients had at least one comorbidity suggest an enhanced severity in the course of the disease.

## Conclusions

In this study we reported different demographic characteristics as well as particularities in systemic cytokines and chemokines signatures between patients from the two initial COVID-19 waves in Argentina. We established that type and concentration of cytokines and chemokines were not associated with the nature and number of comorbidities but rather with the clinical severity and outcome of COVID-19. We also identified immunological and biochemical parameters associated to inflammation that served as prognostic marker within the first but not the second wave. Altogether, our findings provide important information not only at local level by delineating features of the inflammatory/anti-inflammatory response in people from Argentina but also at international level by addressing the impact of comorbidities and the infection wave in cytokine and chemokine production variability upon SARS-CoV-2 infection.

## Data availability statement

The original contributions presented in the study are included in the article/**Supplementary Material**. Further inquiries can be directed to the corresponding authors.

## Ethics statement

The studies involving human participants were reviewed and approved by the “Registro Provincial de Investigación en Salud (RePIS)” (Provincial Registry of Health Research), Córdoba, Argentina under number 4039 and by the Institutional Review Board (IRB) of Hospital Privado Universitario de Córdoba (HPUC), Córdoba, Argentina. The patients/participants provided their written informed consent to participate in this study.

## Author contributions

LA, SCA, NDD, JD, YG, CM-R, CMa, and NEP performed the experiments, analyzed data and prepared the figures. JNQ analyzed data and prepared the figures. DSA supervised experiment and analyzed data. MEV and CMe collaborated with methodology. PI, FMC, and GM analyzed data and discussion of result. MCAV, LC, LSC, LF, PAI, MM, CLM, CCM, MCR-G, and CCS conceived the project and collaborated with the general development of the study. MB, CDA, DE, AK, and JPC performed the recruitment and clinical evaluation of the patients. EVA-R, BAM, AG, and CES conceived the project, designed, supervised and provided overall direction for the study and wrote the manuscript. All authors contributed to the article and approved the submitted version.

## References

[1] Zhu N Zhang D Wang W Li X Yang B Song J. A novel coronavirus from patients with pneumonia in China, 2019. N Engl J Med (2020) 382:727–33. doi: 10.1056/NEJMoa2001017 PMC7092803 31978945

[2] Johns Hopkins Coronavirus Resource Center. Available at: https://coronavirus.jhu.edu/.

[3] Wang D Hu B Hu C Zhu F Liu X Zhang J. Clinical characteristics of 138 hospitalized patients with 2019 novel coronavirus–infected pneumonia in wuhan, China. JAMA. (2020) 323:1061–9. doi: 10.1001/jama.2020.1585 PMC7042881 32031570

[4] Lucas C Wong P Klein J Castro TBR Silva J Sundaram M. Longitudinal analyses reveal immunological misfiring in severe COVID-19. Nature (2020) 584:463–9. doi: 10.1038/s41586-020-2588-y PMC7477538 32717743

[5] Zhou F Yu T Du R Fan G Liu Y Liu Z. Clinical course and risk factors for mortality of adult inpatients with COVID-19 in wuhan, China: a retrospective cohort study. Lancet. (2020) 395:1054–62. doi: 10.1016/S0140-6736(20)30566-3 PMC7270627 32171076

[6] Merad M Blish CA Sallusto F Iwasaki A. The immunology and immunopathology of COVID-19. Science (2022) 375:1122–7. doi: 10.1038/s41586-022-04447-0 PMC12828912 35271343

[7] Zhang Q Bastard P. COVID human genetic effort, cobat a, Casanova J-l. human genetic and immunological determinants of critical COVID-19 pneumonia. Nature (2022) 603:587–98. doi: 10.1038/s41586-022-04447-0 PMC8957595 35090163

[8] Chen Z John Wherry E. T Cell responses in patients with COVID-19. Nat Rev Immunol (2020) 20:529–36. doi: 10.1038/s41577-020-0402-6 PMC7389156 32728222

[9] Ramasamy S Subbian S. Critical determinants of cytokine storm and type I interferon response in COVID-19 pathogenesis. Clin Microbiol Rev (2021) 34:e00299–20. doi: 10.1128/CMR.00299-20 PMC8142516 33980688

[10] Chi Y Ge Y Wu B Zhang W Wu T Wen T. Serum cytokine and chemokine profile in relation to the severity of coronavirus disease 2019 in China. J Infect Dis (2020) 222:746–54. doi: 10.1093/infdis/jiaa363 PMC7337752 32563194

[11] Coperchini F Chiovato L Croce L Magri F Rotondi M. The cytokine storm in COVID-19: An overview of the involvement of the chemokine/chemokine-receptor system. Cytokine Growth Factor Rev (2020) 53:25–32. doi: 10.1016/j.cytogfr.2020.05.003 32446778 PMC7211650

[12] Laing AG Lorenc A Del Molino Del Barrio I Das A Fish M Monin L. A dynamic COVID-19 immune signature includes associations with poor prognosis. Nat Med (2020) 26:1623–35. doi: 10.1038/s41591-020-1038-6 32807934

[13] Casas-Rojo JM Antón-Santos JM Millán-Núñez-Cortés J Lumbreras-Bermejo C Ramos-Rincón JM Roy-Vallejo E. Clinical characteristics of patients hospitalized with COVID-19 in Spain: Results from the SEMI-COVID-19 registry. Rev Clin Esp (Barc) (2020) 220:480–94. doi: 10.1016/j.rce.2020.07.003 PMC7480740 32762922

[14] Guan W Ni Z Hu Y Liang W Ou C He J. Clinical characteristics of coronavirus disease 2019 in China. N Engl J Med (2020) 382:1708–20. doi: 10.1056/NEJMoa2002032 PMC7092819 32109013

[15] Cordova E Mykietiuk A Sued O Vedia LD Pacifico N Hernandez MHG. Clinical characteristics and outcomes of hospitalized patients with SARS-CoV-2 infection in a Latin American country: Results from the ECCOVID multicenter prospective study. PloS One (2021) 16:e0258260. doi: 10.1371/journal.pone.0258260 34624038 PMC8500444

[16] Han H Ma Q Li C Liu R Zhao L Wang W. Profiling serum cytokines in COVID-19 patients reveals IL-6 and IL-10 are disease severity predictors. Emerg Microbes Infect (2020) 9:1123–30. doi: 10.1080/22221751.2020.1770129 PMC7473317 32475230

[17] Del Valle DM Kim-Schulze S Huang H-H Beckmann ND Nirenberg S Wang B. An inflammatory cytokine signature predicts COVID-19 severity and survival. Nat Med (2020) 26:1636–43. doi: 10.1038/s41591-020-1051-9 PMC7869028 32839624

[18] Fabris M Del Ben F Sozio E Beltrami AP Cifù A Bertolino G. Cytokines from bench to bedside: A retrospective study identifies a definite panel of biomarkers to early assess the risk of negative outcome in COVID-19 patients. Int J Mol Sci (2022) 23:4830. doi: 10.3390/ijms23094830 35563218 PMC9101406

[19] Van Damme W Dahake R Delamou A Ingelbeen B Wouters E Vanham G. The COVID-19 pandemic: diverse contexts; different epidemics–how and why? BMJ Glob Health (2020) 5:e003098. doi: 10.1136/bmjgh-2020-003098 PMC7392634 32718950

[20] Sorci G Faivre B Morand S. Explaining among-country variation in COVID-19 case fatality rate. Sci Rep (2020) 10:18909. doi: 10.1038/s41598-020-75848-2 33144595 PMC7609641

[21] Coronavirus. información, recomendaciones y medidas de prevención del ministerio de salud de la nación (2020). Available at: https://www.argentina.gob.ar/salud/coronavirus-COVID-19.

[22] Castro G Sicilia P López L Brbás G Ré V Pisano MB. Vigilancia de variantes de preocupación (VOC) y de interés (VOI) de SARS-CoV-2 en la provincia de córdoba. actualización al 12/08/2021 . Available at: https://repositoriosdigitales.mincyt.gob.ar/vufind/Record/RDUUNC_35f164a3e6279989b33574779de552a5.

[23] Olivero NB Gonzalez-Reiche AS Re VE Castro GM Pisano MB Sicilia P. Phylogenetic analysis and comparative genomics of SARS-CoV-2 from survivor and non-survivor COVID-19 patients in Cordoba, Argentina. BMC Genomics (2022) 23:510–17. doi: 10.1186/s12864-022-08756-6 PMC9282626 35836127

[24] Vigilancia activa de variantes de SARS-CoV-2 en la CABA, provincias de Buenos Aires, Chaco, Entre Ríos, Córdoba, Neuquén y Santa Fe. análisis genómico de casos de variante delta en la argentina. reporte N°27. In: Proyecto argentino interinstitucional de genómica de SARS-CoV2 (2021). Available at: http://pais.qb.fcen.uba.ar/files/reportes/pais-reporte27.pdf.

[25] Saad EJ Barovero MAC Marucco FA Bonazzi STR Barra AT Zlotogora M. Características clínicas y epidemiológicas de pacientes hospitalizados por infección por SARS-CoV-2 en dos hospitales en córdoba: Infección por SARS-CoV-2 en pacientes hospitalizados. Rev Fac Cien Med Cor (2021) 78:303–12. doi: 10.31053/1853.0605.v78.n3.32518 PMC8760912 34617704

[26] Ji D Zhang D Xu J Chen Z Yang T Zhao P. Prediction for progression risk in patients with COVID-19 pneumonia: The CALL score. Clin Infect Dis (2020) 71:1393–9. doi: 10.1111/jop.13114 PMC7184473 32271369

[27] Katz J Yue S. Increased odds ratio for COVID-19 in patients with recurrent aphthous stomatitis. J Oral Pathol Med (2021) 50:114–7. doi: 10.1111/jop.13114 33064856

[28] RStudio. Open source & professional software for data science teams . Available at: https://www.rstudio.com/.

[29] Boehmer TK DeVies J Caruso E van Santen KL Tang S Black CL. Changing age distribution of the COVID-19 pandemic - united states, may-august 2020. MMWR Morb Mortal Wkly Rep (2020) 69:1404–9. doi: 10.15585/mmwr.mm6939e1 PMC7537561 33001872

[30] Jain VK Iyengar KP Vaishya R. Differences between first wave and second wave of COVID-19 in India. Diabetes Metab Syndr (2021) 15:1047–8. doi: 10.1016/j.dsx.2021.05.009 PMC8106236 33992554

[31] Jalali SF Ghassemzadeh M Mouodi S Javanian M Akbari Kani M Ghadimi R. Epidemiologic comparison of the first and second waves of coronavirus disease in babol, north of Iran. Caspian J Intern Med (2020) 11:544–50. doi: 10.22088/cjim.11.0.544 PMC7780865 33425273

[32] Fan G Yang Z Lin Q Zhao S Yang L He D. Decreased case fatality rate of COVID-19 in the second wave: A study in 53 countries or regions. Transbound Emerg Dis (2021) 68:213–5. doi: 10.1111/tbed.13819 32892500

[33] Saito S Asai Y Matsunaga N Hayakawa K Terada M Ohtsu H. First and second COVID-19 waves in Japan: A comparison of disease severity and characteristics. J Infect (2021) 82:84–91. doi: 10.1016/j.jinf.2020.10.033 PMC7605825 33152376

[34] Doidge JC Gould DW Ferrando-Vivas P Mouncey PR Thomas K Shankar-Hari M. Trends in intensive care for patients with COVID-19 in England, Wales, and northern Ireland. Am J Respir Crit Care Med (2021) 203:565–74. doi: 10.1016/S2213-2600(20)30461-6 PMC7924583 33306946

[35] Venkatesan P. The changing demographics of COVID-19. The lancet respiratory medicine Vol. 8. Elsevier (2020). doi: 10.1016/S2213-2600(20)30461-6 PMC7538108 33035468

[36] Kopel J Perisetti A Roghani A Aziz M Gajendran M Goyal H. Racial and gender-based differences in COVID-19. Front Public Health (2020) 8:418. doi: 10.3389/fpubh.2020.00418 32850607 PMC7399042

[37] Richardson S Hirsch JS Narasimhan M Crawford JM McGinn T Davidson KW. Presenting characteristics, comorbidities, and outcomes among 5700 patients hospitalized with COVID-19 in the new York city area. JAMA (2020) 323:2052–9. doi: 10.1001/jama.2020.6775 PMC7177629 32320003

[38] Peckham H de Gruijter NM Raine C Radziszewska A Ciurtin C Wedderburn LR. Male Sex identified by global COVID-19 meta-analysis as a risk factor for death and ITU admission. Nat Commun (2020) 11:6317. doi: 10.1038/s41467-020-19741-6 33298944 PMC7726563

[39] Niessen A Teirlinck AC McDonald SA van der Hoek W van Gageldonk-Lafeber R Knol MJ. Sex differences in COVID-19 mortality in the Netherlands. Infection (2022) 50:709–17. doi: 10.1007/s15010-021-01744-0 PMC9151564 35138581

[40] Wang Y Zhang F Byrd JB Yu H Ye X He Y. Differential COVID-19 symptoms given pandemic locations, time, and comorbidities during the early pandemic. Front Med (2022) 9:770031. doi: 10.3389/fmed.2022.770031 PMC8831795 35155491

[41] Matsunaga N Hayakawa K Asai Y Tsuzuki S Terada M Suzuki S. Clinical characteristics of the first three waves of hospitalised patients with COVID-19 in Japan prior to the widespread use of vaccination: a nationwide observational study. Lancet Reg Health West Pac (2022) 22:100421. doi: 10.1016/j.lanwpc.2022.100421 35300186 PMC8923875

[42] Zeiser FA Donida B da Costa CA Ramos G de O Scherer JN Barcellos NT. First and second COVID-19 waves in Brazil: A cross-sectional study of patients’ characteristics related to hospitalization and in-hospital mortality. Lancet Reg Health Am (2022) 6:100107. doi: 10.1016/j.lana.2021.100107 34746913 PMC8557995

[43] Hernández-Garduño E. Obesity is the comorbidity more strongly associated for covid-19 in Mexico. A case-control study. Obes Res Clin Pract (2020) 14:375–9. doi: 10.1016/j.orcp.2020.06.001 PMC7290168 32536475

[44] Pons MJ Ymaña B Mayanga-Herrera A Sáenz Y Alvarez-Erviti L Tapia-Rojas S. Cytokine profiles associated with worse prognosis in a hospitalized Peruvian COVID-19 cohort. Front Immunol (2021) 12:700921. doi: 10.3389/fimmu.2021.700921 34539631 PMC8440968

[45] Kak G Raza M Tiwari BK. Interferon-gamma (IFN-γ): Exploring its implications in infectious diseases. Biomol Concepts (2018) 9:64–79. doi: 10.1515/bmc-2018-0007 29856726

[46] Bastard P Zhang Q Cobat A Jouanguy E Zhang S-Y Abel L. Insufficient type I IFN immunity underlies life-threatening COVID-19 pneumonia. C R Biol (2021) 344:19–25. doi: 10.5802/crbiol.36 34213846

[47] Zhou Q Chen V Shannon CP Wei X-S Xiang X Wang X. Interferon-α2b treatment for COVID-19. Front Immunol (2020) 11:1061. doi: 10.1038/s41577-020-00429-3 32574262 PMC7242746

[48] Lee JS Shin E-C. The type I interferon response in COVID-19: implications for treatment. Nat Rev Immunol (2020) 20:585–6. doi: 10.1038/s41577-020-00429-3 PMC8824445 32788708

[49] Ling L Chen Z Lui G Wong CK Wong WT Ng RWY. Longitudinal cytokine profile in patients with mild to critical COVID-19. Front Immunol (2021) 12:763292. doi: 10.3389/fimmu.2021.763292 34938289 PMC8685399

[50] Kesmez Can F Özkurt Z Öztürk N Sezen S. Effect of IL-6, IL-8/CXCL8, IP-10/CXCL 10 levels on the severity in COVID 19 infection. Int J Clin Pract (2021) 75:e14970. doi: 10.1111/ijcp.14970 34626520 PMC8646602

[51] Shafiek HK El Lateef HMA Boraey NF Nashat M Abd-Elrehim GAB Abouzeid H. Cytokine profile in Egyptian children and adolescents with COVID-19 pneumonia: A multicenter study. Pediatr Pulmonol (2021) 56:3924–33. doi: 10.1002/ppul.25679 PMC8661994 34536070

[52] Chatterjee S Datey A Sengupta S Ghosh A Jha A Walia S. Clinical, virological, immunological, and genomic characterization of asymptomatic and symptomatic cases with SARS-CoV-2 infection in India. Front Cell Infect Microbiol (2021) 11:725035. doi: 10.3389/fcimb.2021.725035 34993157 PMC8724424

[53] Wang J Yang X Li Y Huang J Jiang J Su N. Specific cytokines in the inflammatory cytokine storm of patients with COVID-19-associated acute respiratory distress syndrome and extrapulmonary multiple-organ dysfunction. Virol J (2021) 18(1):117. doi: 10.1186/s12985-021-01588-y 34088317 PMC8177255

[54] Ramatillah DL Gan SH Pratiwy I Sulaiman SAS Jaber AAS Jusnita N. Impact of cytokine storm on severity of COVID-19 disease in a private hospital in West Jakarta prior to vaccination. PloS One (2022) 17:e0262438. doi: 10.1371/journal.pone.0262438 35077495 PMC8789122

[55] Tang Y Liu J Zhang D Xu Z Ji J Wen C. Cytokine storm in COVID-19: The current evidence and treatment strategies. Front Immunol (2020) 11:1708. doi: 10.3389/fimmu.2020.01708 32754163 PMC7365923

[56] Abidi E El Nekidy WS Alefishat E Rahman N Petroianu GA El-Lababidi R. Tocilizumab and COVID-19: Timing of administration and efficacy. Front Pharmacol (2022) 13:825749. doi: 10.3389/fphar.2022.825749 35250575 PMC8894855

[57] Zhang W Qin C Fei Y Shen M Zhou Y Zhang Y. Anti-inflammatory and immune therapy in severe coronavirus disease 2019 (COVID-19) patients: An update. Clin Immunol (2022) 239:109022. doi: 10.1016/j.clim.2022.109022 35477027 PMC9040414

[58] Rodríguez-Núñez N Gude F Lama A Rábade C Varela A Abelleira R. Health indicators in hospitalized patients with SARS-CoV-2 pneumonia: A comparison between the first and second wave. Arch Bronc (2021) 57:717–9. doi: 10.1016/j.arbres.2021.03.012 PMC7986465 34970016

[59] Meschiari M Cozzi-Lepri A Tonelli R Bacca E Menozzi M Franceschini E. First and second waves among hospitalised patients with COVID-19 with severe pneumonia: a comparison of 28-day mortality over the 1-year pandemic in a tertiary university hospital in Italy. BMJ Open (2022) 12:e054069. doi: 10.1136/bmjopen-2021-054069 PMC8724593 34980623

[60] Gelzo M Scialò F Cacciapuoti S Pinchera B De Rosa A Cernera G. Inducible nitric oxide synthase (iNOS): Why a different production in COVID-19 patients of the two waves? Viruses (2022) 14:534. doi: 10.3390/v14030534 35336941 PMC8948744

[61] Bonnet B Cosme J Dupuis C Coupez E Adda M Calvet L. Severe COVID-19 is characterized by the co-occurrence of moderate cytokine inflammation and severe monocyte dysregulation. EBioMed (2021) 73:103622. doi: 10.1016/j.ebiom.2021.103622 PMC8526358 34678611

[62] Cabaro S D’Esposito V Di Matola T Sale S Cennamo M Terracciano D. Cytokine signature and COVID-19 prediction models in the two waves of pandemics. Sci Rep (2021) 11:20793. doi: 10.1038/s41598-021-00190-0 34675240 PMC8531346

[63] Khedar RS Mittal K Ambaliya HC Mathur A Gupta JB Sharma KK. Greater covid-19 severity and mortality in hospitalized patients in second (Delta variant) wave compared to the first: Single centre prospective study in India. medRxiv (2021). doi: 10.1101/2021.09.03.21263091. Preprint.

[64] Gisondi P Piaserico S Naldi L Dapavo P Conti A Malagoli P. Incidence rates of hospitalization and death from COVID-19 in patients with psoriasis receiving biological treatment: A northern Italy experience. J Allergy Clin Immunol (2021) 147:558–560.e1. doi: 10.1016/j.jaci.2020.10.032 33160968 PMC7644231

[65] Damiani G Pacifico A Bragazzi NL Malagoli P. Biologics increase the risk of SARS-CoV-2 infection and hospitalization, but not ICU admission and death: Real-life data from a large cohort during red-zone declaration. Dermatol Ther (2020) 33:e13475. doi: 10.1111/dth.13475 32356577 PMC7261990

[66] Bigdelou B Sepand MR Najafikhoshnoo S Negrete JAT Sharaf M Ho JQ. COVID-19 and preexisting comorbidities: Risks, synergies, and clinical outcomes. Front Immunol (2022) 13:890517. doi: 10.3389/fimmu.2022.890517 35711466 PMC9196863

[67] De Ciuceis C Rossini C La Boria E Porteri E Petroboni B Gavazzi A. Immune mechanisms in hypertension. High Blood Press Cardiovasc Prev (2014) 21:227–34. doi: 10.1007/s40292-014-0040-9 24446309

[68] Daryabor G Atashzar MR Kabelitz D Meri S Kalantar K. The effects of type 2 diabetes mellitus on organ metabolism and the immune system. Front Immunol (2020) 11:1582. doi: 10.3389/fimmu.2020.01582 32793223 PMC7387426

[69] Pedersen SF Ho Y-C. SARS-CoV-2: a storm is raging. J Clin Invest (2020) 130:2202–5. doi: 10.1172/jci137647 PMC7190904 32217834

[70] Ye Q Wang B Mao J. The pathogenesis and treatment of the `Cytokine storm’ in COVID-19. J Infect (2020) 80:607–13. doi: 10.1016/j.jinf.2020.03.037 PMC7194613 32283152

[71] Wauters E Van Mol P Garg AD Jansen S Van Herck Y Vanderbeke L. Discriminating mild from critical COVID-19 by innate and adaptive immune single-cell profiling of bronchoalveolar lavages. Cell Res (2021) 31:272–90. doi: 10.1038/s41422-020-00455-9 PMC8027624 33473155

[72] Virdis A Taddei S. How to evaluate microvascular organ damage in hypertension. High Blood Press Cardiovasc Prev (2011) 18:163–7. doi: 10.2165/11593630-000000000-00000 22283670

[73] Ruhl L Pink I Kühne JF Beushausen K Keil J Christoph S. Endothelial dysfunction contributes to severe COVID-19 in combination with dysregulated lymphocyte responses and cytokine networks. Sig Transduct Target Ther (2021) 6:1–15. doi: 10.1038/s41392-021-00819-6 PMC8661333 34893580

[74] Amraei R Rahimi N. COVID-19, renin-angiotensin system and endothelial dysfunction. Cells MDPI (2020) 9:1652. doi: 10.3390/cells9071652 PMC7407648 32660065

