# Supplementary material for: Different cytokine and chemokine profiles in hospitalized patients with COVID-19 during the first and second outbreaks from Argentina show no association with clinical comorbidities
Source: Front Immunol. 2023 Feb 1;14:1111797. doi: 10.3389/fimmu.2023.1111797 (PMC9929547; doi:10.3389/fimmu.2023.1111797)
Supplement: Supplementary file 1 [file DataSheet_1.pdf]

# Supplemental Figure 1

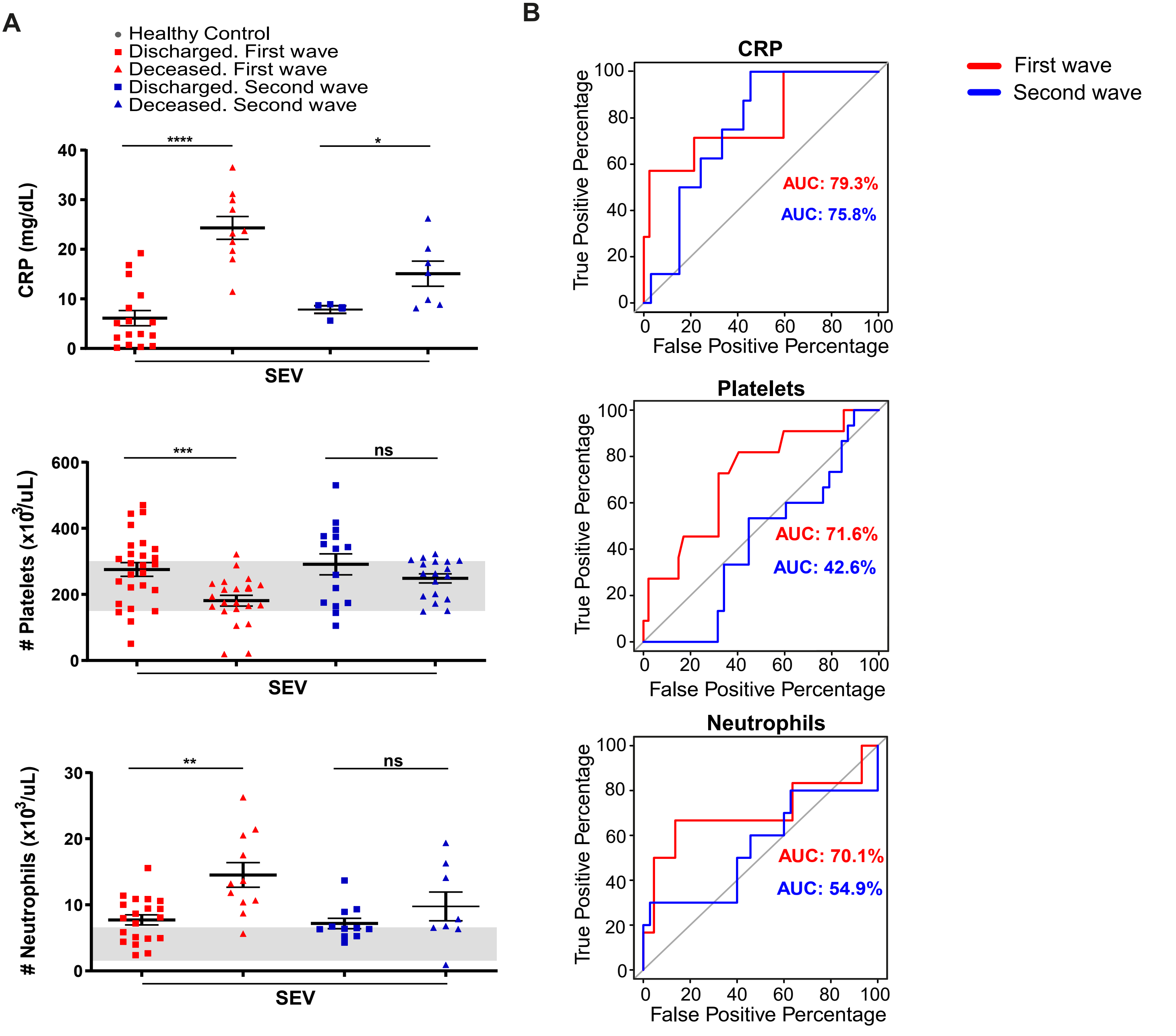


**Figure S1**

Supplemental Figure 1: Differences in biochemical markers in hospitalized patients with severe COVID-19 that present different clinical outcomes. A) Values of CRP, platelets and neutrophils counts determined in samples from HC (gray) and from discharged and deceased severe COVID-19 patients recruited during first (red) and second (blue) waves. Scatter plots show the individual measurements (dots) and black line shows the concentration mean of each analyt. For statistical analyses Unpaired t test with Welch's correction was used (***P < 0.001, **P < 0.01 and *P < 0.05; ns, not significant). Shaded areas indicate normal reference ranges for each marker. B) ROC curve of CRP levels and platelets and neutrophils counts in deceased versus discharged severe COVID-19 patients from the first (blue lines) and the second (red lines) waves. Values of AUC for each wave are shown with different color code indicated in the figure.
